# Research on the Consensus Convergence Rate of Multi-Agent Systems Based on Hermitian Kirchhoff Index Measurement

**DOI:** 10.3390/e27101035

**Published:** 2025-10-02

**Authors:** He Deng, Tingzeng Wu

**Affiliations:** School of Mathematics and Statistics, Qinghai Minzu University, Xining 810007, China; denghe0529@163.com

**Keywords:** Hermitian Kirchhoff index, multi-agent system, hybrid network, consensus convergence rate

## Abstract

Multi-agent systems (MAS) typically model interaction topologies using directed or undirected graphs when analyzing consensus convergence rates. However, as system complexity increases, purely directed or undirected networks may be insufficient to capture interaction heterogeneity. This paper adopts hybrid networks as interaction topology to investigate strategies for improving consensus convergence rates. We propose the Hermitian Kirchhoff index, a novel metric based on resistance distance, to quantify the consensus convergence rates and establish its theoretical justification. We then examine how adding or removing edges/arcs affects the Hermitian Kirchhoff index, employing first-order eigenvalue perturbation analysis to relate these changes to algebraic connectivity and its associated eigenvectors. Numerical simulations corroborate the theoretical findings and demonstrate the effectiveness of the proposed approach.

## 1. Introduction

The consensus problem of multi-agent systems (MAS) is a fundamental issue in coordinated control, focusing on the design of distributed protocols that enable all agents to reach agreement on a common state. This problem has attracted sustained attention owing to its wide range of applications [1,2,3,4,5,6]. Interaction topologies are typically modeled by directed or undirected networks, and agent dynamics are characterized by consensus protocols. For first-order MAS with single-integrator dynamics, consensus is achieved if and only if the communication graph contains a directed spanning tree [7], with the consensus convergence rate determined by the algebraic connectivity of the network (the second smallest eigenvalue of the Laplacian matrix, $\lambda_{2}$) [8]. For second-order systems with double-integrator dynamics, all agents must synchronize both position and velocity [9]. While the spanning-tree condition remains relevant for directed networks [7], undirected networks typically require the graph to be connected [10]; directed networks additionally require constraints on the real and imaginary parts of the Laplacian eigenvalues [11]. The consensus convergence rate is influenced by both $\lambda_{2}$ and the spectral radius (the largest eigenvalue $\lambda_{N}$).

Since maximizing algebraic connectivity is an NP-hard problem [12] and optimal topology design is subject to constraints, researchers have proposed various topology-optimization strategies to enhance convergence performance, such as edge addition [13,14], edge reorganization [15], and weight adjustment [16,17]. Notably, communication links are not universally beneficial: some are labeled “redundant links” [18], since their existence can waste energy, bandwidth, and computational resources and even shorten system lifetime, particularly in battery-constrained sensor networks [19]. Under specific conditions, removing such links can preserve or even improve the consensus convergence rate. However, identifying redundant links is challenging. On the one hand, existing methods can be computationally expensive, for example greedy algorithms based on the synchronization metric $\lambda_{2}/\lambda_{N}$ [20]. On the other hand, analyses that rely on diffusion importance or particular graph families (e.g., pinwheel graphs) lack generality [21], which limits their applicability across different scenarios. Moreover, removing edges in undirected networks may increase the network diameter, which may contradict the intuition that smaller diameters accelerate convergence in directed networks [22]. Leader–follower MAS is an important variant: because the leader has no in-degree, the Laplacian matrix becomes asymmetric and eigenvalues may be complex, complicating efforts to improve algebraic connectivity. Studies have also shown that adding reverse arcs to directed or weighted chains can reduce algebraic connectivity, highlighting the nonlinear impact of adjusting communication links on performance [14].

Modern real-world networks have grown increasingly complex, and hybrid networks have become prevalent. They arise in MAS [23,24], biology [25], and social systems [26]. However, most research on MAS consensus problems focuses on purely directed or undirected interaction topologies, with limited exploration of hybrid networks. Moreover, Chen et al. [18] employed algebraic connectivity in undirected networks to identify redundant links and thereby improve the consensus convergence rate. Gao et al. [14] used a generalized algebraic connectivity in a special directed setting—the leader–follower multi-agent system—to investigate methods for improving consensus convergence. Because hybrid networks lack a direct analogue of algebraic connectivity, new measurement methods based on matrix-theoretic tools are required, for example the magnetic Laplacian [27] and the Hermitian adjacency matrix [28]. In this regard, Lin et al. [29] constructed the Hermitian resistance matrix and the Hermitian Kirchhoff index from the eigenvalues and eigenvectors of the Hermitian Laplacian to assess connectivity and robustness of hybrid networks. Given the relationship between the Hermitian Kirchhoff index and algebraic connectivity, a natural question arises: can the Hermitian Kirchhoff index serve as a metric for the consensus convergence rate of multi-agent systems whose interaction topology is a hybrid network?

This paper proposes using the Hermitian Kirchhoff index to measure the consensus convergence rate of multi-agent systems with hybrid networks as interaction topologies, based on Hermitian matrix theory, and employs rigorous mathematical methods to establish its relationship with algebraic connectivity. Meanwhile, to optimize such multi-agent systems, it explores the impact of adding or removing edges/arcs in the interaction topology on the Hermitian Kirchhoff index and summarizes the resulting rules. The rest of this paper is organized as follows. Section 1 introduces the application background and research status of multi-agent systems. Section 2 presents the definition of the Hermitian matrix and the Hermitian Kirchhoff index. In Section 3, we discuss the relationship between the Hermitian Kirchhoff index and algebraic connectivity, and explain its rationality as a metric for measuring the consensus convergence rate. Section 4 investigates the effect of adding edges/arcs on the Hermitian Kirchhoff index. Section 5 examines the effect of removing edges/arcs on the Hermitian Kirchhoff index. Section 6 conducts numerical simulations on the theoretical results from the above sections to validate their correctness and effectiveness. Finally, Section 7 concludes the paper.

## 2. Preliminaries

Let *G* be a hybrid network with a finite set of vertices V(G) and a subset E(G)⊆V(G)×V(G), and then the edge set E(G) is the union of undirected edges and directed edges. For convenience, we define undirected edges as $v_{i}↔v_{j}$, and directed edges as $v_{i}\to v_{j}$ if the direction is from $v_{i}$ to $v_{j}$. The diagonal matrix $D=diag(d_{1},d_{2},\ldots ,d_{n})$ is the degree matrix, where $d_{i}$ is the degree of $v_{i}$ of the underlying graph Ω(G). We denote $H^{*}={\left(\bar{H}\right)}^{T}$. If $H^{*}=H$, then *H* is a Hermitian matrix. A Hermitian adjacency matrix of a hybrid network is represented by the matrix $ℵ\in C^{V\times V}$ [30], whose elements are as follows:

$$\begin{array}{c} \eta_{ij}=\left\{\begin{array}{c} 1,\;\;\;\;\;\;\mathrm{if}\;v_{i}↔v_{j};\\ i,\;\;\;\;\;\;\;\mathrm{if}\;v_{i}\to v_{j};\\ -i,\;\;\;\;\mathrm{if}\;v_{i}\leftarrow v_{j};\\ 0,\;\;\;\;\;\;\mathrm{otherwise}. \end{array}\right. \end{array}$$

In the network *G*, the value of the mixed walk $T=v_{1}v_{2}\ldots v_{k}$ is $\eta \left(T\right)=\eta_{12}\eta_{23}\ldots \eta_{(k-1)k}$. When η(T)=1 (η(T)=−1), the mixed walk is positive (negative).

**Lemma** **1** (Yu et al. [31])**.** *A hybrid network G is positive if and only if for any two vertices $v_{1}$ and $v_{2}$, all mixing paths from $v_{1}$ to $v_{2}$ have the same value.*

**Lemma** **2** (Yu et al. [32])**.** *Let G be a connected hybrid network with vertices $T=v_{1},v_{2},\ldots ,v_{n}$. If $L_{ℵ}=D-ℵ$ is a singular positive semidefinite matrix, then 0 is a simple eigenvalue with an eigenvector:*

$$\begin{array}{c} 1_{ℵ}={\left(1,\bar{\eta (T_{2})},\bar{\eta (T_{3})},\ldots ,\bar{\eta (T_{n})}\right)}^{T}, \end{array}$$

*where $T_{i}$ is the 1−i-walk in G.*

The eigenvalues and orthogonal eigenvectors of the Hermitian Laplacian matrix are represented by $\lambda_{i}$ and $u_{i}={\left(u_{i1},u_{i2},\ldots ,u_{in}\right)}^{T}$, respectively. The unitary matrix is represented by

$$\begin{array}{c} U_{ℵ}=\left(u_{1},u_{2},\ldots ,u_{n}\right), \end{array}$$

where $u_{n}=\left(1/\sqrt{n}\right)1_{ℵ}$.

Then, we have

$$\begin{array}{c} U_{ℵ}^{*}L_{ℵ}U_{ℵ}=diag\left[\lambda_{1},\ldots ,\lambda_{n-1},0\right], \end{array}$$

and

$$\begin{array}{c} U_{ℵ}^{*}U_{ℵ}=U_{ℵ}U_{ℵ}^{*}=I, \end{array}$$

$$\begin{array}{c} \sum_{k=1}^{n}u_{ki}\bar{u_{kj}}=\sum_{k=1}^{n}\bar{u_{ik}}u_{jk}=\left\{\begin{array}{c} 1,\;\;\;\;\;\;\mathrm{if}\;i=j;\\ 0,\;\;\;\;\;\;\mathrm{if}i\neq \;j. \end{array}\right. \end{array}$$

**Lemma** **3** (Liu et al. [28])**.** *Assume G is a hybrid network. Then the following are equivalent:*

1. *G is positive;*
2. *$G∽G_{u}$.*

**Lemma** **4** (Yu et al. [31])**.** *Assume G is a positive hybrid network. Then the following statement holds:*

1. *$SP_{ℵ}\left(G\right)=SP_{A}\left(G_{u}\right)$, where $SP_{A}\left(G_{u}\right)$ is the spectrum of the adjacency matrix of $G_{u}$;*
2. *$SP_{L_{ℵ}}\left(G\right)=SP_{L}\left(G_{u}\right)$, where $SP_{L}\left(G_{u}\right)$ is the spectrum of the Laplacian matrix of $G_{u}$.*

**Lemma** **5** (Lin et al. [29])**.** *Let G be a positive hybrid network with n vertices. The Hermitian Kirchhoff index can be expressed as*

$$\begin{array}{c} Kf_{H}=\sum_{i<j}\eta \left(T_{i}\right)\bar{\eta (T_{j})}r_{ℵ}\left(i,j\right)=\sum_{i<j}r\left(i,j\right)=n\sum_{k=1}^{n-1}\frac{1}{\lambda_{k}}, \end{array}$$

*where $r_{ℵ}\left(i,j\right)$ is the resistance distance of the Hermitian Laplace matrix, and r(i,j) is the resistance distance of the underlying network $G_{u}$.*

**Lemma** **6** (Lin et al. [29])**.** *Assume G is a general hybrid network with n vertices, $L_{ℵ}$ is the Hermitian Laplace matrix, $L_{ℵ}^{†}$ is the matrix obtained by the Moore-Penrose generalized inverse of $L_{ℵ}$, and the Hermitian Kirchhoff index can be expressed as*

$$\begin{array}{c} Kf_{H}=\sum_{i<j}r_{ℵ}\left(i,j\right)=\sum_{i<j}{\left(L_{ℵ}^{†}\right)}_{ii}+{\left(L_{ℵ}^{†}\right)}_{jj}-{\left(L_{ℵ}^{†}\right)}_{ij}-{\left(L_{ℵ}^{†}\right)}_{ji}, \end{array}$$

*where $r_{ℵ}\left(i,j\right)$ is the resistance distance of the Hermitian Laplace matrix.*

In continuous time, a first-order multi-agent system (MAS) can be described by Laplacian dynamics. For multi-agent systems with hybrid networks as interaction topology, each agent’s state $x_{i}\left(t\right)$ satisfies

$${\dot{x}}_{i}\left(t\right)=\sum_{j\subseteq N_{i}}\eta_{ij}\left(x_{j}\left(t\right)-x_{i}\left(t\right)\right),\;\;\;\;\;\;\;\;\;\;\;\;i=1,\ldots ,n,$$

It can also be expressed in the form of a matrix, e.g., $\dot{x}=-L_{ℵ}x$. Whether the system reaches consensus (i.e., all agent states converge to the same value) depends on the connectivity of the interaction topology and the weight distribution. For general complex-weighted adjacency matrices *A*, theoretical results indicate that the Laplace matrix L=D−A has a simple zero eigenvalue and the corresponding steady-state consensus subspace is only one-dimensional if and only if the graph contains a spanning tree and is “balanced,” meaning $a_{ij}a_{ji}\geq 0$ for each edge and no conflicting cycles exist [7]. In particular, since directed edges are weighted by ±i for Hermitian matrix, the condition $a_{ij}a_{ji}={\left|a_{ij}\right|}^{2}\geq 0$ is automatically satisfied. As long as the underlying graph is connected and contains no “negative cycle” (i.e., the graph is balanced), $L_{ℵ}$ has a unique zero eigenvalue and all other eigenvalues are positive. Therefore, when the mixed network contains a (weighted) spanning tree, the first-order Laplacian dynamics $\dot{x}=-L_{ℵ}x$ converges to consensus.

The consensus convergence rate is determined by the smallest nonzero eigenvalue or its real part of the underlying network. Additional several classic convergence rate indices are widely used, including the network coherence index and synchronizability measures, which are also constructed from Laplacian eigenvalues. For instance, the steady-state covariance of consensus dynamics under noise is tightly related to the sum of the inverses of all nonzero Laplacian eigenvalues. In fact, the Kirchhoff index (sum of effective resistance distances) has been linked to robustness of first-order consensus protocols under stochastic disturbances for undirected networks [33]. This highlights that beyond algebraic connectivity, the full spectrum (e.g., the sum of eigenvalue reciprocals) also captures convergence and robustness properties.

## 3. Relationship Between Hermitian Kirchhoff Index and Algebraic Connectivity

In this section, we will explain and prove the relationship between the Hermitian Kirchhoff index and algebraic connectivity.

For undirected networks, the Kirchhoff index is

$$\begin{array}{c} Kf_{H}=n\sum_{k=2}^{n}\frac{1}{\lambda_{k}}=n\cdot trace\left(L^{†}\right), \end{array}$$

where $L^{†}$ is the inverse of the matrix. Because the eigenvalue multiplicity of the Laplacian matrix of undirected network is the number of connected components (denoted as *c*),

$$\begin{array}{c} Kf_{H}=n\sum_{k=c+1}^{n}\frac{1}{\lambda_{k}}, \end{array}$$

In an undirected network, algebraic connectivity is the second smallest eigenvalue $\lambda_{2}$ (the smallest non-zero eigenvalue). In hybrid networks, algebraic connectivity is defined as the smallest non-zero eigenvalue $\lambda_{\min}$, as the eigenvalue corresponding to 0 is determined by the vectors corresponding to the connected components. To establish the relationship between $Kf_{H}$ and algebraic connectivity, we distinguish between positive and general hybrid networks: positive hybrid networks have $Kf_{H}$ index expressions consistent with their underlying networks, while general hybrid networks use Moore-Penrose generalized inverses for resistance distance, differing slightly from undirected networks.

**Theorem** **1.** *For a connected mixed network G with n vertices and m edges, its Hermitian Kirchhoff index $Kf_{H}$ and algebraic connectivity λ satisfy the following relationship:*

$$\begin{array}{c} \frac{n{\left(n-1\right)}^{2}}{2m+\left(n-1\right)\lambda_{\min}}\leq Kf\leq \frac{n(n-1)}{\lambda_{\min}} \end{array}$$

**Proof.**  For the lower bound, according to Rayleigh’s principle, the algebraic connectivity can be expressed as

λmin=minx≠0x⊥1ℵx*Lℵxx*x

In undirected networks, there is a known inequality:

$$\begin{array}{c} Kf=n\sum_{k=2}^{n}\frac{1}{\lambda_{k}}\leq \frac{n(n-1)}{\lambda_{\min}} \end{array}$$

However, the eigenvalues of the Hermitian Laplace matrix of the hybrid network are all non-negative real numbers. The eigenvalues of the Moore-Penrose generalized inverse $L^{†}$ are the reciprocals of the non-zero eigenvalues of the original matrix. The zero eigenvalue corresponds to zero. If the zero eigenvalue multiplicity is 1, the Hermitian Kirchhoff index of the general hybrid network is

$$\begin{array}{cc} Kf_{H} & =\sum_{i<j}r_{ℵ}\left(i,j\right)\\ & =\sum_{i<j}\sum_{k=2}^{n}\frac{1}{\lambda_{k}}{\left|u_{ki}-u_{kj}\right|}^{2}\\ & =\frac{1}{2}\sum_{i,j}\sum_{k=2}^{n}\frac{1}{\lambda_{k}}{\left|u_{ki}-u_{kj}\right|}^{2}\\ & =\frac{1}{2}\sum_{k=2}^{n}\frac{1}{\lambda_{k}}\left(2n-2{\left|\sum_{i=1}^{n}u_{ki}\right|}^{2}\right)\\ & =n\sum_{k=2}^{n}\frac{1}{\lambda_{k}}-\sum_{k=2}^{n}\frac{1}{\lambda_{k}}{\left|\sum_{i=1}^{n}u_{ki}\right|}^{2}. \end{array}$$

For the eigenvector $u_{k}$ with non-zero eigenvalue $\lambda_{k}>0$, it is orthogonal to the zero eigenvector $u_{0}$, that is, $u_{k}^{*}u_{0}=0$. If $u_{0}$ is a constant vector, then

$$\sum_{i=1}^{n}u_{ki}=0⟹{\left|\sum_{i=1}^{n}u_{ki}\right|}^{2}=0,$$

This result is the same as the $Kf_{H}$ expression of the positive hybrid network, but when $u_{0}$ is not a constant, since we are considering the random assignment of edge directions with probability *p* in the Erdös-Rényi hybrid network, the direction assignment satisfies weak correlation, so when n→∞,

$$E\left[{\left|\sum_{i=1}^{n}u_{ki}\right|}^{2}\right]=O\left(\frac{1}{n}\right)\to 0,$$

Thus there is

$$Kf_{H}\approx n\sum_{k=2}^{n}\frac{1}{\lambda_{k}}=n\cdot tr\left(L^{†}\right),$$

Using the Cauchy–Schwarz inequality for the harmonic mean of eigenvalues:

$$\begin{array}{c} \sum_{k=2}^{n}\frac{1}{\lambda_{k}}\geq \frac{{\left(n-1\right)}^{2}}{\sum_{k=2}^{n}\lambda_{k}}, \end{array}$$

Since $\lambda_{k}\geq \lambda_{\min}$ for k≥2, then:

$$\begin{array}{c} \sum_{k=2}^{n}\lambda_{k}\geq \left(n-1\right)\lambda_{\min}, \end{array}$$

Since

$$\begin{array}{c} \sum_{k=2}^{n}\frac{1}{\lambda_{k}}\geq \frac{{\left(n-1\right)}^{2}}{\sum_{k=2}^{n}\lambda_{k}}=\frac{{\left(n-1\right)}^{2}}{2m}, \end{array}$$

Combined with $\sum_{k=2}^{n}\lambda_{k}\geq \left(n-1\right)\lambda_{\min}$, we have

$$\begin{array}{cc} \frac{{\left(n-1\right)}^{2}}{2m} & =\frac{{\left(n-1\right)}^{2}}{2m+\left(n-1\right)\lambda_{\min}}\cdot \frac{2m+\left(n-1\right)\lambda_{\min}}{2m}\\ & \geq \frac{{\left(n-1\right)}^{2}}{2m+\left(n-1\right)\lambda_{\min}}. \end{array}$$

Finally, we obtain

$$\begin{array}{c} Kf_{H}=n\sum_{k=2}^{n}\frac{1}{\lambda_{k}}\geq \frac{n{\left(n-1\right)}^{2}}{2m+\left(n-1\right)\lambda_{\min}}, \end{array}$$

For the upper bound, according to the monotonicity of the eigenvalues, $\lambda_{\min}\leq \lambda_{3}\leq \ldots \leq \lambda_{n}$, we have

$$\begin{array}{c} \frac{1}{\lambda_{k}}\leq \frac{1}{\lambda_{\min}}\;\;\;\;\;\;\;\;\forall k\geq 2, \end{array}$$

Thus

$$\begin{array}{c} \sum_{k=2}^{n}\frac{1}{\lambda_{k}}\leq \sum_{k=2}^{n}\frac{1}{\lambda_{\min}}=\frac{n-1}{\lambda_{\min}}, \end{array}$$

Multiply both sides by *n* to get

$$\begin{array}{c} Kf_{H}=n\sum_{k=2}^{n}\frac{1}{\lambda_{k}}\leq \frac{n(n-1)}{\lambda_{\min}}, \end{array}$$

The proof is complete. □

From the above results, we can see that although there is no concise calculation expression for the $Kf_{H}$ index, its calculation essentially depends on the inverse $\frac{1}{\lambda_{k}}$ of all non-zero eigenvalues $\lambda_{k}$ ($\lambda_{k}>0$), and $\lambda_{\min}$ is the smallest non-zero eigenvalue, and $\sum_{k=2}^{n}\frac{1}{\lambda_{k}}$ is mainly dominated by $\frac{1}{\lambda_{\min}}$, that is, if $\lambda_{\min}$ is very small (resulting in slow convergence), then $\frac{1}{\lambda_{\min}}$ will be very large, which will significantly increase the value of $Kf_{H}$; conversely, a large $\lambda_{\min}$ (fast convergence) Corresponding to a small $\frac{1}{\lambda_{\min}}$, it tends to produce a smaller $Kf_{H}$ value. In consensus analysis, it plays a role similar to the Kirchhoff index in undirected networks, which is used to characterize robustness against additive noise. As the sum of the “effective resistance” of all node pairs, the increase of $Kf_{H}$ intuitively means that the network connectivity efficiency is reduced and the information propagation resistance is increased, which is bound to be unfavorable for fast convergence. Therefore, for a connected hybrid network, the size of $Kf_{H}$ can still strongly indicate the speed of the consistent convergence rate: a large $Kf_{H}$ indicates slow convergence, and a small one indicates fast convergence. In summary, the first-order MAS consensus analysis based on the Hermitian Laplace matrix not only continues the framework of traditional Laplace dynamics, but also introduces a new network index—the Hermitian Kirchhoff index. This index has advantages in describing network connectivity and robustness in the case of hybrid networks.

## 4. On the Effect of Adding a Few Edges/Arcs for the ${\mathrm{Kf}}_{H}$ Index

In this section, we analyze the effect of adding a few edges/arcs on the $Kf_{H}$ index with connected hybrid networks.

**Theorem** **2** (Influence of Adding Directed Arcs)**.** *Let $v={\left(v_{1},\ldots ,v_{n}\right)}^{T}$ be the unit eigenvector corresponding to the smallest non-zero eigenvalue $\lambda_{\min}$ of $L_{ℵ}$. After adding the directed arc Arc=(p→r), the $Kf_{H}$ exponential change of the new network is:*

$$\delta Kf_{H}=-n\sum_{k=2}^{n}\frac{\delta \lambda_{k}}{\lambda_{k}^{2}}\approx -\frac{n}{\lambda_{\min}^{2}}\delta \lambda_{\min},\;where\;\delta \lambda_{\min}={\left|v_{p}\right|}^{2}+{\left|v_{r}\right|}^{2}-2ℑ\left(v_{p}^{*}v_{r}\right).$$

and $\delta \lambda_{\min}>0$ always holds true regardless of the circumstances, then $Kf_{H}$ decreases, where ℑ represents the imaginary component of the complex number, and $v_{p}^{*}$ is the conjugate complex number of $v_{p}$.

**Proof.**  Add the correction matrix of the arc $e=(v_{p}\to v_{r})$:

$$R_{\mathrm{arc}}=\underset{\mathrm{Degree}\;\mathrm{increment}}{\underset{︸}{e_{p}e_{p}^{T}+e_{r}e_{r}^{T}}}-\underset{\mathrm{Adjacency}\;\mathrm{increment}}{\underset{︸}{\left(ie_{r}e_{p}^{T}-ie_{p}e_{r}^{T}\right)}},$$

Eigenvalue perturbation formula:

$$\delta \lambda_{\min}=v^{H}R_{\mathrm{arc}}v,$$

Expand calculation:

$$\begin{array}{cc} v^{H}R_{\mathrm{arc}}v & =v^{H}\left(e_{p}e_{p}^{T}\right)v+v^{H}\left(e_{r}e_{r}^{T}\right)v-iv^{H}\left(e_{r}e_{p}^{T}\right)v+iv^{H}\left(e_{p}e_{r}^{T}\right)v\\ & =\;{\left|v_{p}\right|}^{2}+{\left|v_{r}\right|}^{2}-i\left(v_{r}^{*}v_{p}\right)+i\left(v_{p}^{*}v_{r}\right)\\ & =\;{\left|v_{p}\right|}^{2}+{\left|v_{r}\right|}^{2}+i\left(v_{p}^{*}v_{r}-v_{r}^{*}v_{p}\right)\\ & =\;{\left|v_{p}\right|}^{2}+{\left|v_{r}\right|}^{2}-2ℑ\left(v_{p}^{*}v_{r}\right). \end{array}$$

where $ℑ\left(v_{p}^{*}v_{r}\right)=a_{p}b_{r}-b_{p}a_{r}$, by complex division:

$$ℑ\left(\frac{v_{r}}{v_{p}}\right)=ℑ\left(v_{r}v_{p}^{-1}\right)=\frac{ℑ(v_{p}^{*}v_{r})}{|v_{p}{|}^{2}},$$

When $ℑ\left(\frac{v_{r}}{v_{p}}\right)<0$, obviously $\delta \lambda_{\min}>0$; when $ℑ\left(\frac{v_{r}}{v_{p}}\right)>0$, from $|v_{p}{|}^{2}+|v_{r}{|}^{2}\geq 2\left|v_{p}v_{r}\right|$ and phase optimization, we can obtain $\delta \lambda_{\min}>0$. Thus $\delta Kf_{H}<0$ is always valid under any conditions. □

**Theorem** **3** (Influence of Adding Undirected Edges)**.** *Adding an undirected edge {q,t} is equivalent to adding two reverse arcs. The eigenvalue changes to*

$$\delta \lambda_{\min}={\left|v_{q}\right|}^{2}+{\left|v_{t}\right|}^{2}-2ℜ\left(v_{q}^{*}v_{t}\right),$$

*And $\delta \lambda_{\min}>0$ always holds true regardless of the circumstances, $Kf_{H}$ decreases, where ℜ represents the real part of the complex number.*

**Proof.**  Undirected edge correction matrix:

$$R_{\mathrm{edge}}=e_{q}e_{q}^{T}+e_{t}e_{t}^{T}-e_{q}e_{t}^{T}-e_{q}e_{t}^{T},$$

According to the eigenvalue perturbation formula, we have

$$\begin{array}{cc} \delta \lambda_{\min} & =v^{H}R_{\mathrm{edge}}v\\ & =\;{\left|v_{q}\right|}^{2}+{\left|v_{t}\right|}^{2}-v^{H}\left(e_{t}e_{q}^{T}\right)v-v^{H}\left(e_{q}e_{t}^{T}\right)v\\ & =\;|v_{q}{|}^{2}+{\left|v_{t}\right|}^{2}-v_{q}^{*}v_{t}-v_{t}^{*}v_{q}\\ & =\;|v_{q}{|}^{2}+{\left|v_{t}\right|}^{2}-2ℜ\left(v_{q}^{*}v_{t}\right). \end{array}$$

Since

$$ℜ\left(\frac{v_{q}}{v_{t}}\right)=ℜ\left(v_{q}v_{t}^{-1}\right)=\frac{ℜ(v_{q}^{*}v_{t})}{|v_{t}{|}^{2}},$$

When $ℜ\left(\frac{v_{q}}{v_{t}}\right)<0$, obviously $\delta \lambda_{\min}>0$; when $ℜ\left(\frac{v_{p}}{v_{r}}\right)>0$, from $ℜ\left(v_{q}^{*}v_{t}\right)<\frac{|v_{q}{|}^{2}+{\left|v_{t}\right|}^{2}}{2}$ and the phase difference satisfies this condition, we can obtain $\delta \lambda_{\min}>0$. Thus $\delta Kf_{H}<0$ is always valid under any conditions. □

**Theorem** **4** (Mixing Optimization)**.** *After adding the edge set $E_{\mathrm{undir}}$ and the arc set $E_{\mathrm{dir}}$, the total eigenvalue changes:*

$$\delta \lambda_{\min}=\sum_{\left\{q,t\right\}\in E_{\mathrm{undir}}}\left(|v_{q}{|}^{2}+{\left|v_{t}\right|}^{2}-2ℜ\left(v_{q}^{*}v_{t}\right)\right)+\sum_{\left(p\to r\right)\in E_{\mathrm{dir}}}\left(|v_{p}{|}^{2}+{\left|v_{r}\right|}^{2}-2ℑ\left(v_{p}^{*}v_{r}\right)\right),$$

*If the total phase contribution is*

$$\Delta =\sum_{\left\{q,t\right\}}ℜ\left(\frac{v_{q}}{v_{t}}\right)+\sum_{\left(p\to r\right)}ℑ\left(\frac{v_{r}}{v_{p}}\right)>0,$$

*Then $\delta \lambda_{\min}>0$ and Kf decreases.*

**Proof.**  The total perturbation matrix $R=\sum R_{\mathrm{edge}}+\sum R_{\mathrm{arc}}$, is obtained by linear combination and Theorems 5 and 6:

$$\begin{array}{cc} \delta \lambda_{\min} & =v^{H}Rv\\ & =\sum v^{H}R_{\mathrm{edge}}v+\sum v^{H}R_{\mathrm{arc}}v\\ & =\sum_{q,t}\left(\right|v_{q}{|}^{2}+|v_{t}{|}^{2}-2ℜ\left(v_{q}^{*}v_{t}\right))+\sum_{p\to r}\left(\right|v_{p}{|}^{2}+|v_{r}{|}^{2}-2ℑ\left(v_{p}^{*}v_{r}\right)). \end{array}$$

When Δ>0, the perturbations work together to make $\delta \lambda_{\min}>0$, $\delta Kf_{H}<0$. □

**Corollary** **1** (Optimal Directed Arcs Selection)**.** *Fix the head vertex $v_{r}$, select m tail vertices from the candidate set S to maximize $Kf_{H}$ reduction: Choose $v_{p}$ to maximize $ℑ\left(\frac{v_{p}}{v_{r}}\right)$.*

**Corollary** **2** (Optimal Undirected Edges Selection)**.** *Select an edge e={q,t} from the candidate set that maximizes $Kf_{H}$ reduction: Choose an edge that maximizes $ℜ\left(\frac{v_{q}}{v_{t}}\right)$.*

**Proof.**  Since $\delta Kf_{H}\propto -\delta \lambda_{\min}$ and $\delta \lambda_{\min}$ is proportional to the phase term, maximizing the phase contribution is to maximize the $Kf_{H}$ reduction. □

## 5. On the Effect of Removing a Few Edges/Arcs for the ${\mathrm{Kf}}_{H}$ Index

The removal operation can be viewed as the inverse of the addition operation: removing the directed arc e=(p→r) is equivalent to adding a negative perturbation $-R_{\mathrm{arc}}$; removing the undirected edge {u,v} is equivalent to adding a negative perturbation $-R_{\mathrm{edge}}$.

**Theorem** **5** (Inverse Property of Eigenvalue Perturbation for Removal)**.** *Assume that the eigenvalue $\lambda_{\min}$ of $L_{ℵ}$ corresponds to the unit eigenvector v, and the perturbation matrix corresponding to the edge deletion operation is −R, then the first-order eigenvalue changes:*

$$\delta \lambda_{\min}=-v^{H}Rv,$$

*where R is the perturbation matrix when adding this element.*

**Proof.**  From the eigenvalue perturbation theory:

$$\delta \lambda_{\min}=v^{H}\left(-R\right)v=-v^{H}Rv,$$

This is strictly true for Hermitian matrices, and $L_{ℵ}$ is a Hermitian matrix. □

**Theorem** **6** (Influence of Removing Directed Arcs)**.** *Let v be the $\lambda_{\min}$ corresponding eigenvector of $L_{ℵ}$, after removing the arc Arc=(p→r):*

$$\delta \lambda_{\min}=-\left(\right|v_{p}{|}^{2}+|v_{r}{|}^{2})+2ℑ\left(v_{p}^{*}v_{r}\right),$$

*and $\delta \lambda_{\min}<0$ always holds true regardless of the circumstances, then $Kf_{H}$ increases.*

**Proof.**  From the perturbation result of adding arcs and Theorem 2, we have

$$v^{H}R_{\mathrm{arc}}v=|v_{p}{|}^{2}+{\left|v_{r}\right|}^{2}-2ℑ\left(v_{p}^{*}v_{r}\right),$$

Substituting into Theorem 5:

$$\delta \lambda_{\min}=-\left(|v_{p}{|}^{2}+{\left|v_{r}\right|}^{2}-2ℑ\left(v_{p}^{*}v_{r}\right)\right),$$

Phase condition analysis:

$$\begin{array}{ccc} \delta \lambda_{\min}<0 & \Leftrightarrow |v_{p}{|}^{2}+{\left|v_{r}\right|}^{2}-2ℑ\left(v_{p}^{*}v_{r}\right)>0\\ ℑ(v_{p}^{*}v_{r}) & ≃ℑ\left(\frac{v_{r}}{v_{p}}\right) & , \end{array}$$

When $ℑ\left(\frac{v_{r}}{v_{p}}\right)<0$, obviously $\delta \lambda_{\min}<0$ is established; when $ℑ\left(\frac{v_{r}}{v_{p}}\right)>0$, from $|v_{p}{|}^{2}+|v_{r}{|}^{2}\geq 2\left|v_{p}v_{r}\right|$ and phase optimization, we can obtain indirectly $\delta \lambda_{\min}<0$. Thus $\delta Kf_{H}>0$ is always valid under any conditions. □

**Theorem** **7** (Influence of Removing Undirected Edges)**.** *After removing the edge {q,t}:*

$$\delta \lambda_{\min}=-\left(\right|v_{q}{|}^{2}+|v_{t}{|}^{2})+2ℜ\left(v_{q}^{*}v_{t}\right),$$

*And $\delta \lambda_{\min}<0$ always holds true regardless of the circumstances, then $Kf_{H}$ increases.*

**Proof.**  From the perturbation result of adding edges and Theorem 3, we can get

$$v^{H}R_{\mathrm{edge}}v=|v_{q}{|}^{2}+{\left|v_{t}\right|}^{2}-2ℜ\left(v_{q}^{*}v_{t}\right),$$

Substituting into Theorem 5:

$$\delta \lambda_{\min}=-\left(|v_{q}{|}^{2}+{\left|v_{t}\right|}^{2}-2ℜ\left(v_{q}^{*}v_{t}\right)\right),$$

By the addition theorem:

$$|v_{q}{|}^{2}+{\left|v_{t}\right|}^{2}-2ℜ\left(v_{q}^{*}v_{t}\right)>0,$$

Therefore, $\delta \lambda_{\min}<0$, $Kf_{H}$ increases. □

**Theorem** **8** (Connectivity Maintenance Conditions)**.** *Necessary and sufficient conditions for the network to remain connected after removing elements:*

$$\lambda_{\min}\left(G\setminus e\right)>0$$

*In this case, $Kf_{H}$ is finite and computable. If $\lambda_{\min}\left(G\setminus e\right)=0$, then $Kf_{H}\to \infty$.*

**Proof.**  From the Hermitian Laplacian property, we know that $\lambda_{\min}>0$ if and only if the network is connected. If the network is not connected after removal, then the null space dimension of $L_{ℵ}>1$, which will cause $L_{ℵ}^{†}$ to be undefined or $r_{ℵ}\left(i,j\right)\to \infty$, in which case $Kf_{H}$ does not exist or $Kf_{H}\to \infty$. □

Theoretical investigations in Section 4 and Section 5 examine the mechanism by which adjustments of edges/arcs can improve the first-order MAS’s convergence performance. The conclusions are strictly derived from first-order eigenvalue perturbation theory and therefore apply only to the small-perturbation regime in which a few edges/arcs are added or removed at any one time. We emphasize that the present study is restricted to a small number of operations for reasons of both theoretical rigor and practical feasibility. If a large number of edge/arc modifications are performed continuously, the network topology may change substantially and the core assumptions underlying our analysis no longer hold. Specifically, (1) the phase-discrimination criterion depends on the pre-operation eigenvector v; subsequent modifications induce eigenvector drift, so decisions based on an outdated eigenvector will accumulate error and may fail; (2) large-scale changes can cause eigenvalue reordering, i.e., the eigenmode associated with the algebraic connectivity $\lambda_{\min}$ may switch, rendering the preceding analysis inapplicable; (3) removal operations in particular can readily destroy network connectivity. Once $\lambda_{\min}=0$, the Hermitian Kirchhoff index $Kf_{H}$ diverges and the derived formulas lose meaning.

Therefore, restricting the scope of this study to a small number of operations should be regarded not as a limitation but as a deliberate choice made for theoretical rigor. This restriction ensures that conclusions about the effects of individual operations are precise and reliable within the small-perturbation framework. More importantly, a deep understanding of single-operation effects establishes a solid foundation for the optimization of network topology at larger scales. A practical and feasible strategy is to incorporate the results of this work into an iterative optimization scheme: at each iteration, perform the single optimal operation determined by the current network state, update the network, and repeat the process. In this way, the present analysis provides the essential theoretical basis and core decision criteria for efficient large-scale topology-optimization algorithms.

## 6. Numerical Simulation

In this section, we will use several numerical examples to illustrate the theoretical results in the previous sections.

**Figure 1:**
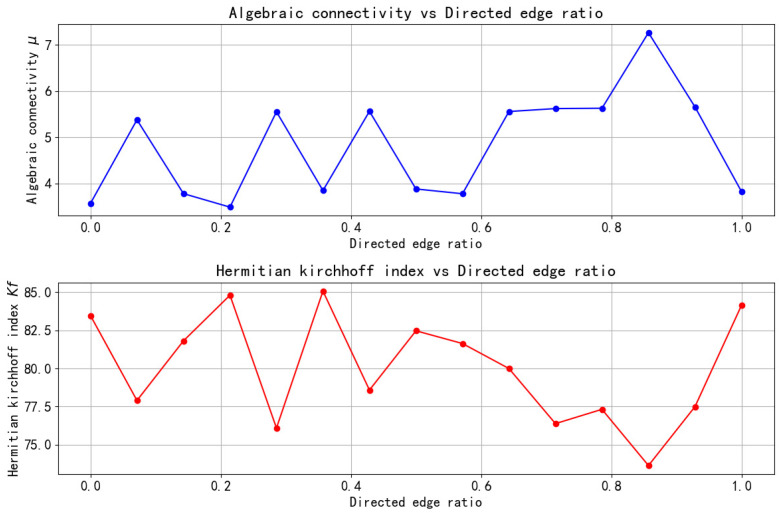
The relationship between Hermitian Kirchhoff index and algebraic connectivity.

**Example** **1.** *To verify the relationship between the $Kf_{H}$ index and algebraic connectivity, we generated a random mixed network with 30 vertices and 100 edges, tested 15 different gradients of directed edge proportion used as the independent variable and constructed line graphs of the $Kf_{H}$ index and algebraic connectivity, as shown in Figure 1.*

From Figure 1, we can see that the trends of the $Kf_{H}$ index and the algebraic connectivity are exactly opposite. With the increase in the proportion of directed edges, the algebraic connectivity develops in a cyclical trend of “first increase and then decrease”, while the $Kf_{H}$ index develops in a cyclical trend of “first decrease and then increase”, which corresponds to it. It is also reflected in some turning points. Therefore, this further confirms that the $Kf_{H}$ index is negatively correlated with the algebraic connectivity. As the $Kf_{H}$ index increases, the algebraic connectivity will decrease. Therefore, it is feasible to use the $Kf_{H}$ index to replace the algebraic connectivity as an indicator to measure the consistent convergence rate of the multi-agent system.

**Figure 2:**
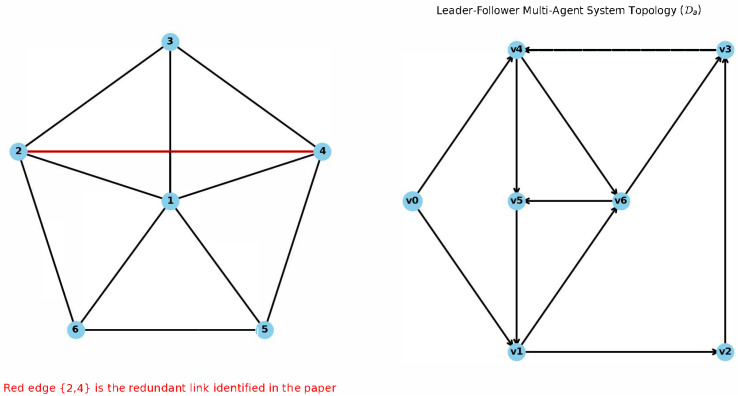
Directed and undirected graph examples. Communication Network $G_{a}$ of a First-Order MAS (Figure 6 in Gao et al. [14]).

**Example** **2.** *To further verify the relationship between the $Kf_{H}$ index and algebraic connectivity, we adopt the existing numerical simulation examples of Gao et al. [14] and Lin et al. [18], as shown in Figure 2. Starting with an undirected network, the Laplacian eigenvalue of $G_{a}$ is (0, 2.3820, 3.3820, 4.6180, 5.6180, 6), $\lambda_{\min}\left(G_{a}\right)=2.3820$, and the original $Kf_{H}=7.6596$. After removing the non-redundant edge 1, 2, $\lambda_{\min}\left(G_{a}\right)$ decreases, while $Kf_{H}$ increases. For the directed network, the algebraic connectivity $\lambda_{\min}\left(D_{a}\right)=0.401$, and the original $Kf_{H}=42.714$. After adding arc $\left(V_{1}\to V_{5}\right)$, $\lambda_{\min}\left(D_{a}\right)$ increases, and $Kf_{H}$ decreases. When arcs $\left(V_{2}\to V_{5}\right)$ and $\left(V_{3}\to V_{5}\right)$ are added respectively, $\lambda_{\min}\left(D_{a}\right)$ decreases and $Kf_{H}$ increases, which is consistent with Theorem 2.*

**Figure 3:**
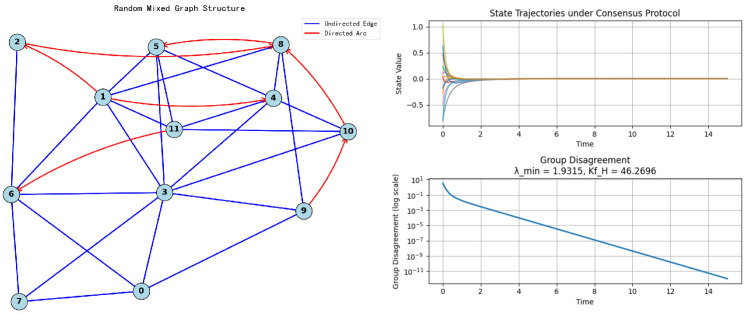
Random hybrid network structure and state trajectory of nodes.

**Figure 4:**
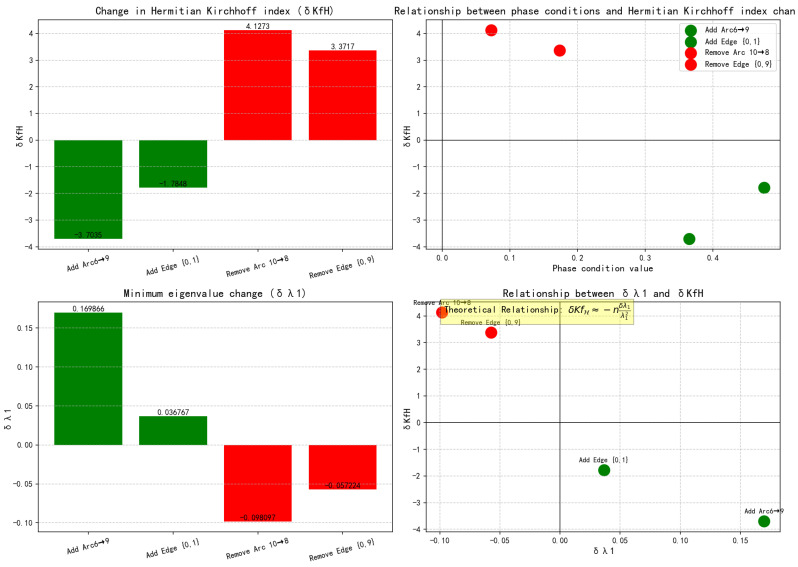
Visualize the impact of operations.

**Example** **3.** *To verify whether the conditions for the influence of adding or removing edges or arcs on the $Kf_{H}$ index are consistent, we use the Erdös–Rényi hybrid network as example to perform numerical simulation. At the same time, we also show the state trajectory of all nodes corresponding to hybrid network with topology shown in Figure 3. We can see that over time, the state values of all nodes gradually converge to a consistent value, and the group inconsistency also decays exponentially and gradually approaches 0. The convergence rate and decay rate are directly proportional to the algebraic connectivity, which means they are inversely proportional to the $Kf_{H}$ index. In other words, as time goes by, the greater the algebraic connectivity, the smaller $Kf_{H}$, the faster the convergence rate and the faster the decay rate. Through calculation, we can obtain the minimum non-zero eigenvalue $\lambda_{\min}=0.6955$ and $Kf_{H}=16.1172$ of the original Hermitian Laplace matrix $L_{ℵ}$. After a series of operations such as adding or removing edges/arcs, the results are shown in Table 1 and Figure 4.*

From Table 1 we can see that whether adding edges/arcs or removing edges/arcs, the predicted changes and the actual $\delta Kf_{H}$ change trends are exactly the same. When adding edges/arcs, the phase condition is positive, and the actual $\delta \lambda_{\min}$ is also positive—this in turn leads to a negative actual $\delta Kf_{H}$, indicating that when adding edges/arcs, the phase condition is positively correlated with $\delta \lambda_{\min}$ and negatively correlated with $\delta Kf_{H}$; when removing edges/arcs, the phase condition is positive, and the actual $\delta \lambda_{\min}$ is negative. At this time, the actual $\delta Kf_{H}$ is positive, indicating that when removing edges/arcs, the phase condition is negatively correlated with $\delta \lambda_{\min}$ and positively correlated with $\delta Kf_{H}$. This relationship is more clearly shown in Figure 4, which is consistent with the previous theoretical results, indicating that the previous theory is correct and effective.

## 7. Summary

For MAS with hybrid network as interactive topology, this paper first proposes the $Kf_{H}$ index based on resistance distance as an indicator to measure the consistent convergence rate, and discusses its relationship with algebraic connectivity, demonstrating its rationality as such an indicator. Then, the influence of adding or removing edges/arcs on the $Kf_{H}$ index is explored. By introducing the eigenvalue perturbation matrix, this paper studies the change of the corresponding network matrix independently, and establishes its relationship with algebraic connectivity and its eigenvectors, and the phase condition for judging the change of the $Kf_{H}$ index is obtained: when adding or removing arcs, the imaginary part of the ratio of the head end to the tail end needs to be calculated, and when adding or removing edges, the real part of the ratio of the head end to the tail end need to be calculated. By comparing the change in algebraic connectivity, the results are extended to adding edges and arcs at the same time, which can be extended to the scenario of adding edges and arcs simultaneously, covering all tail and head vertices of these arcs. These results can be used to improve the consistent convergence rate of MAS.

Future research should consider the case where the interaction topology of MAS is a weighted mixed graph, or consider the effect of a large number of adjustments to edges/arcs on $Kf_{H}$ index.

## Figures and Tables

**Table 1:** The impact of operations on Hermitian Kirchhoff index.

Operation	Phase Condition	Predicting Change	Actual $\delta \lambda_{\min}$	Actual $\delta {\mathrm{Kf}}_{H}$
Add Arc 6→9	0.3651	$Kf_{H}$ decrease	0.1699	−3.7035
Add Edge {0,1}	0.4758	$Kf_{H}$ decrease	0.0368	−1.7848
Remove Arc 10→8	0.0724	$Kf_{H}$ increase	−0.0981	4.1273
Remove Edge {0,9}	0.1737	$Kf_{H}$ increase	−0.0572	3.3717

## Data Availability

The original contributions presented in this study are included in the article. Further inquiries can be directed to the corresponding author.
